# “My Body Hates Me”: A Qualitative Analysis of the Experience of Functional Nausea in Adolescent Girls and Their Mothers

**DOI:** 10.3390/children7080083

**Published:** 2020-07-26

**Authors:** Michelle A. T. Cole, Dima Qu’d, Marcus G. Wild, Alexandra C. Russell, Aimee R. Caillet, Amanda L. Stone

**Affiliations:** 1 Department of Medicine, Health, and Society, Vanderbilt University, Nashville, TN 37212, USA; michelle.a.cole@vanderbilt.edu; 2 Department of Anesthesiology, Vanderbilt University Medical Center, Nashville, TN 37212, USA; dima.qud@vumc.org; 3 Department of Psychology, Vanderbilt University, Nashville, TN 37212, USA; marcus.wild@vanderbilt.edu; 4 Division of Pediatric Gastroenterology, Department of Pediatrics, Vanderbilt University Medical Center, Nashville, TN 37212, USA; alexandra.c.russell@vumc.org (A.C.R.); aimee.r.caillet@vumc.org (A.R.C.)

**Keywords:** qualitative, somatic symptoms, functional gastrointestinal disorders, chronic nausea, pediatric

## Abstract

Nausea is a somatic sensation typically associated with the need to vomit in order to remove a toxin from the body. When nausea occurs in the absence of a specific structural cause or toxin, it is classified as a functional gastrointestinal disorder (FGID). Functional nausea was newly recognized in 2016 as a FGID in children and little is known about its prevalence, course or patient experiences. Nausea co-occurring with functional abdominal pain in childhood has been associated with long-term risk for anxiety and ongoing somatic symptoms into young adulthood. However, few studies have focused uniquely on the experience and impact of nausea on youth. The present study aimed to qualitatively understand the experiences of adolescent girls with functional nausea and their parents. Five mother–daughter dyads were recruited from a specialized pediatric gastroenterology clinic focused on nausea and completed semi-structured interviews. Interviews were transcribed and coded using interpretive phenomenological analysis (IPA). Four main themes emerged: nausea interference, body frustration, misunderstanding of symptoms, and maternal helplessness and guilt. These themes were similar to prior studies on the experiences of youth with chronic pain but also indicated unique challenges due to nausea, such as significant food restriction and subsequent weight loss.

## 1. Introduction

Nausea is a subjective experience of discomfort which evolutionarily has served a protective purpose to warn an individual of a potential toxic ingestion [1,2]. However, in some individuals, nausea becomes chronic and no longer serves this adaptive purpose. The Rome IV classification system for functional gastrointestinal disorders (FGIDs) defines functional nausea in children and adolescents as bothersome nausea (a) occurring at least twice per week for at least two months as the primary symptom experienced, (b) not typically accompanied by vomiting, (c) not usually associated with meals, (d) not better explained by another diagnosis [3]. Functional nausea is a clinical diagnosis that is made after relevant evaluation for other possible underlying organic causes, such as inflammatory bowel disease [4], gastroparesis [5], eosinophilic esophagitis [6], celiac disease [7], and gastritis [8], has been reassuring. 

Historically, functional nausea was only considered a diagnosis for adults, but was added as a pediatric diagnosis in 2016 [3]. Because functional nausea is a newly classified pediatric diagnosis, prevalence data in pediatric populations are difficult to pinpoint. One large, school-based study found that approximately 23% of healthy adolescents experienced weekly nausea [9]. In adolescents with pain-related FGIDs, approximately one-half to two-thirds also experienced bothersome nausea at least two times per week [10,11]. Indeed, most studies to date on functional nausea in youth have evaluated nausea in the context of pediatric functional abdominal pain, one of the more closely related pediatric diagnoses. Adolescents who experienced both functional abdominal pain and chronic nausea reported a lower quality of life as well as heightened levels of anxiety, compared to adolescents with only functional abdominal pain [10]. 

The experience of functional nausea could be similar to the experience of functional abdominal pain in that both are persistent somatic experiences without visible or biochemical abnormalities to explain them. The “brain-gut axis” refers to the connection between emotional experience and dysregulation in the brain and the somatic experiences of the body [12]. As biological symptoms are experienced in the gut as a result of possible imbalances in the gut microbiome, hypersensitivities to digestive processes and other mechanisms, signals are communicated from the gut to the brain. The brain’s interpretations of such symptoms can interact with anxiety and maladaptive coping strategies and lead to an increase in symptoms, thus, the relationship is bidirectional [13]. Children who experience chronic nausea also experience headaches, fatigue and increased anxiety, with up to 70% of adolescent girls with functional nausea also reporting symptoms of anxiety [9]. Anxiety perpetuates nausea, and nausea can worsen anxiety; this cyclical psychological distress can increase the experience of somatic symptoms [14]. 

Similar to pain, researchers have developed self-report assessment tools designed to quantitatively capture nausea severity in pediatric populations. Some of the tools developed to quantify nausea severity include the Nausea Severity Scale (NSS) and the Baxter Animated Retching Face (BARF) scale [15,16]. However, these tools do not fully capture the lived experiences of youth with functional nausea. Indeed, no studies, to our knowledge, have qualitatively evaluated the experience of functional nausea in youth to identify key themes that can guide research and clinical practice.

As a somatic symptom and FGID, functional nausea is likely best understood in the context of the biopsychosocial model [17]. This three-fold model incorporates biological factors (e.g. the physical sensations of nausea resulting from activity of the gastrointestinal system), psychological factors (e.g., anxiety or worry related to symptoms), and social factors (e.g., impact of family systems and peers on nausea) to form a more complete understanding of the underlying mechanisms and lived experience of functional nausea. Studies in youth with pain-related FGIDs that have evaluated the impact of symptoms on parental well-being, as well as parent responses to children’s symptoms, have found associations among parental psychological distress, parental protective responses to children’s pain, and children’s functional impairment [18,19,20,21,22]. The experience of functional nausea in youth likely extends to parents, as parents are the gatekeepers for health care, driving children to appointments, advocating for their child’s needs and assuming caregiving responsibilities at home. 

Although the experiences of adolescents with chronic pain and their parents could be extrapolated to the experiences of adolescents with other chronic somatic symptoms, such as nausea, it is important to understand how these experiences could differ. In a longitudinal study of youth with functional abdominal pain, nausea predicted poor health outcomes in late adolescence and young adulthood above and beyond the effects of pain [23]. Thus, it is important to further understand the subjective experience of nausea specifically and how nausea impacts adolescents’ quality of life.

Our study aimed to qualitatively describe the experience of functional nausea in adolescent girls and their mothers through semi-structured interviews. We chose to focus specifically on adolescent females, because nausea and FGIDs are more common in women, and the lived experience of nausea in adolescents may differ based on gender [24]. Further, in line with a biopsychosocial model, we aimed to describe the impact of pediatric functional nausea on mothers. 

## 2. Method

### 2.1. Participants 

Five females ranging from ages 11 to 17 (Mean = 14.6, SD = 2.06) and their mothers participated in the study. Eligibility criteria were female gender, between 11 and 17 years of age, and lack of organic diseases that could have a confounding effect on the data. Adolescents were excluded if they had a primary language other than English or had an organic disease that could better explain the nausea, such as inflammatory bowel disease (IBD). For three of the five adolescents, functional nausea was the only diagnosis. Two of the five adolescents also had a history of irritable bowel syndrome. The majority of adolescents in the study had below average to average BMI (median: 19.84 kg/m^2^, range: 17.41–29.02 kg/m^2^). All participants identified as White (100%, *n* = 5) and all of the parents enrolled were mothers. On average, mothers were 43 years old (SD = 5.10 years), and maternal education ranged from “some college/associates degree” to “master’s degree.” 

All adolescents were patients referred to the Nausea Clinic at Vanderbilt University Children’s Hospital because nausea constituted their primary presenting gastrointestinal symptom. All adolescents were diagnosed with functional nausea by a pediatric gastroenterologist and had undergone diagnostic testing (e.g., endoscopy, gastric emptying study when indicated). In many cases, a pediatric gastroenterologist within the same practice referred the adolescent to the specialty nausea clinic due to their nausea persistence and severity. The Nausea Clinic takes an integrative medicine approach for treating functional nausea in children [25] and is staffed by a pediatric gastroenterologist with advanced training in integrative medicine and a supervised doctoral student in clinical psychology. 

### 2.2. Procedure

All study procedures were approved by the Vanderbilt University Institutional Review Board # 191069. The study included two recruitment procedures. First, the primary physician for the pediatric nausea clinic asked potentially eligible patients if they were interested in the study. The majority of the sample was recruited through this method (80%). Second, the clinic mailed letters to adolescents and parents who had attended the nausea clinic within the six months prior to the start of recruitment for the study to ask if they would be willing to participate. One participant was recruited through this method (20%).

Upon receiving permission from the family, a research assistant described the study, screened the patient for eligibility, and collected demographic information. For eligible patients, the research assistant then completed informed consent and assent forms with the dyad. 

Immediately following consent/assent, the research assistant conducted semi-structured qualitative interviews with each of the adolescents and a parent (Table 1). Some questions were adapted from a similar qualitative study in youth with chronic pain [26]. Interviews were conducted separately for each adolescent and parent to ensure the privacy and independence of responses. Adolescents and parents were asked to wait in another room or the clinic waiting room while the other completed their interview. The semi-structured interviews asked a series of open-ended questions to aid in the understanding of adolescents and their parents’ experiences with chronic nausea. The interviews also included probe questions for adolescents and parents who struggled to relay their experiences to the interviewer. The interviews lasted between 15 and 30 min, and were audio recorded. Dyads were compensated with a USD 20 gift card for participation.

### 2.3. Data Analysis 

Interpretative phenomenological analysis (IPA) was used to analyze the data. IPA is a method of analyzing qualitative data that examines a phenomenon and seeks to understand, or interpret, the experiences of the phenomenon by first reading thoroughly through individual interviews, then looking for common themes amongst a collection of interviews [27]. Foundational to IPA is the belief that verbally expressed ideas are reflective of cognitive beliefs. It is a qualitative method of analysis that differs from quantitative psychological research by employing a focus on inductive generation of themes from detailed examinations of individuals to understand the experiences of psychological phenomena. 

As an inductive methodology, researchers utilizing IPA do not begin with a clear hypothesis, but rather try to put aside their own ideas and conceptions to understand how the participants make sense of their condition, and create theories based on this understanding. This is difficult to do because of inherent biases and the natural tendency to create narrative explanations for our observations. The goal, however, is to minimize these biases and tendencies in order for the interview data to speak for itself. The phenomenon in this study is functional nausea. The semi-structured interviews conducted allowed participants to share their experiences in a deep and meaningful manner through guided questions and probes. Interviews were recorded and transcribed in order to understand how the participants experience and cope with their chronic nausea. The goal of IPA is not to understand the experiences of the participants from the researcher’s perspective, but to analyze how the participants understand and narrate their own experiences [28]. IPA is also unique in that it does not try to find evidence for an already existing theory, but rather uses the data to find new themes [19]. Smith, the creator of IPA, writes that “the meaning individuals ascribe to events should be of central concern to the social scientist, but also that those meanings are only obtained through a process of interpretation” [29]. 

A small number of participants were included in the study because of the desire for detailed interviews, which would provide a more meaningful understanding of the phenomena of nausea. Parents were also included in the study to achieve a broader perspective on how the experiences of chronic nausea also affect the familial system. In coding the data and looking for themes, transcribed interviews were re-read several times, looking first for individual experiences, and then common narratives expressed from both the adolescents and their parents. Larger themes and smaller themes were recorded based on significance and repetition throughout the transcripts. Two independent coders (M.C., D.Q.) reviewed the transcripts and identified themes. Coders met with a third individual (A.S.) to decide on the final themes. Researchers were cognizant of returning to the transcript to ensure the accurate portrayal of the participants’ cognitions and understandings of their own experiences. 

## 3. Results 

From the interviews conducted, four superordinate themes were identified from the adolescent girls and their mothers. These themes were (1) nausea’s interference with day-to-day functioning, (2) body frustration, (3) misunderstanding of symptoms, and (4) maternal helplessness and guilt. Pseudonyms are employed here to protect the confidentiality of the participants. 

### 3.1. Nausea Interference 

Adolescents and their mothers both noted that nausea significantly interfered with daily activities and functioning. For many, this interference was multi-faceted and resulted in impaired functioning across multiple domains, including physical activity, social interactions, mood, and eating behaviors. Adolescents noted a significant discrepancy between their current physical functioning due to nausea and their desired activity levels.

*“I can’t always do something I wanna do because I feel so nauseous and sick.” (Louise)*

*“I can’t necessarily do a lot of the things that normal teens my age would do like going outside and exercising and running around and goofing off” (Sarah)*

Adolescents described the impact of their nausea as unpredictable and intermittent, noting that some days they would feel okay and be able to participate in activities, but other days, nausea would leave them completely debilitated.

*“I’ll wake up so sick to my stomach that I cannot go to school…because there’d be sometimes that like it was so bad that I would literally not be able to get out of bed.” (Julia)*

Mothers similarly noted these changes in their daughters’ activity levels and functioning, attributing these shifts to the effects of nausea on overall vitality.

*“She’s not as energetic or her full self because she used to be really outgoing and wanted to do everything, and now she’s always on the couch, wrapped in a blanket because she doesn’t feel good, so her daily activities and hanging out with friends has decreased a lot.” (Julia’s mother)*

Mothers observed changes in their daughters’ personality and mood because of the chronic nature of nausea. This was perceived as a significant change or loss by mothers and daughters, noting that nausea took away positive features of their personality (e.g., outgoingness, confidence, energy).

*“… it tends to make you feel a little more down or irritated, it’s kind of hard to watch or engage in a conversation… you get those mood shifts, and if you’re usually funny, it’s hard to be funny, and you’re just like more upset.” (Sarah)*

*“She’s normally a very confident girl and you know can handle whatever you put in front of her but [nausea] just shakes her to the core...it just takes away all of herself assuredness and leaves her feeling so vulnerable” (Ann’s mother)*

Because adolescents perpetually felt sick due to nausea, they adopted behaviors consistent with having an illness like a stomach virus, such as isolating and withdrawing from others, resting, and avoiding eating and mealtimes. Adolescents struggled to adapt to their chronic nausea and noted that nausea increased their anxiety and left them feeling unsure of how to move forward and engage in activities of daily living. Some noted the cyclical nature of anxiety, where their anxiety may spike because of a stressor unrelated to nausea, then their nausea would increase, which, in turn, would increase their anxiety even further.

*“Nausea makes the anxiety happen but stress activates the nausea.” (Louise)*

*“My anxiety will spike my nausea and my nausea will spike my anxiety to an anxiety attack level.” (Sarah)*

In addition to impact of nausea on social activities and mood, adolescents described nausea and nausea-related anxiety as interfering with eating and mealtime behaviors. Adolescents expressed that they desired to be able to eat like a typical teenager, but that nausea often made them modify their diet or significantly restrict foods.

*“There’ll be some things that I really want to eat, but I’m just like I know I’ll get sick from it…”(Julia)*

*“Simple sugars like candy really makes me nauseous, fruits with a lot of sugars in them can make me nauseous, acidic foods, things that are spicy, dairy. I’m cutting out everything, like red meats and heavily processed foods, too like…if it’s a highly processed thing, it tends to not settle as well, things with like breads and stuff kind of feels like you’re eating a wet sponge…and it just kind of like soaks up everything and inflates and it makes you feel really heavy...I just cut out all the food groups.” (Emily)*

These restrictions, in turn, led to additional concerns regarding weight and whether adolescents were taking in enough calories to maintain an appropriate weight for their age. The need to eat and maintain weight despite perpetual feelings of nausea added an additional layer of anxiety for adolescents and their mothers.

*“I don’t eat as much and then I’ll be kind of worried that I’m not eating enough” (Louise)*

Nausea further interfered with family meal times. Some mothers described modifying their own behaviors related to meal preparation to accommodate their daughter’s nausea. Others described changing expectations regarding family meal times, allowing adolescents to skip the family meal because of nausea.

*“I tend to cater the whole meal towards what she’ll eat.” (Ann’s mother)*

*“We don’t really have any family dinners cuz I don’t sit at the table and eat…I just kind of sit there.” (Sarah)*

Overall, mother–daughter dyads described nausea as interfering across a number of life domains including eating and mealtimes, social interactions, personality, mood, and physical functioning. Many adolescents and mothers described distress regarding these changes, longing for a return to the way life used to be before the nausea started. The broad impact of nausea left many feeling like nausea dominated and took over their daily lives. Louise’s mother summarized this when she stated “it (nausea) affects everything all the time.”

### 3.2. Body Frustration

Adolescents described feeling especially frustrated with their body and the inability of their body to eat and process food normally. Perpetually feeling sick and nauseous, some adolescents desired to switch bodies with someone else or have a different stomach so that they could resume activities and feel like an average teenager.

*“Sometimes I don’t like my body as much because it can make me feel like this and I kind of wanna escape from my body and from all this nausea.” (Louise)*

Adolescents’ descriptions of dissatisfaction and frustration towards their body often focused on aspects of the body’s functionality, such as the ability to process food and maintain weight. This frustration with their body’s functioning at times generalized and affected other aspects of their mood, leading to feelings of depression.

*“There for a while I was like really down about myself…I was like kind of depressed about it because I was like, my body hates me and stuff.” (Julia)*

For some adolescents, this frustration with their body’s functioning extended to their body’s appearance. Because of the inability to eat due to nausea, adolescents described significant weight loss and changes in their body’s appearance that left them and others feeling concerned.

*“I’m definitely not happy with [my body] because even though I’m naturally skinny, I know that I’m unhealthy with weight- that I am underweight… I remember I didn’t really think about my body before…I was tall and I was skinny, and that’s fine. I had the muscle I needed when I was younger…I was healthy, it was all good.” (Sarah)*

The response to changes in appearance due to nausea-associated weight loss was primarily one of dissatisfaction and frustration. Adolescents longed for their body to return to its function and appearance before they started experiencing nausea. Although adolescents as a whole largely reported dissatisfaction with their appearance due to nausea-associated weight loss, one mother reported concerns that her daughter sees the thinness as a positive. Other mothers expressed concerns regarding their daughters’ weight loss, but did not express similar concerns regarding increased body image as a positive consequence of the weight loss.

### 3.3. Misunderstanding of Symptoms

Adolescents shared that they felt misunderstood by others in their experiences of nausea. Several teens chose not to share about their nausea with friends because they worried about what others might think or had negative experiences with sharing about their symptoms in the past. Actual or forecasted misinterpretations of chronic nausea included teenage pregnancy, eating disorders, excessive complaining, and being perceived as weird or different.

*“Most of the time when a teenage girl says she’s nauseous all the time, they take it into context as she’s pregnant.” (Sarah)*

*“Some just back away when I tell them that I’m sick to my stomach. They’re just like ‘oh that’s weird’” (Julia)*

In addition to peers, family members and physicians also expressed misunderstanding or dismissed adolescents’ symptoms. At times, adolescents attributed this misunderstanding to a lack of empathy or knowledge of nausea. Because nausea is invisible and adolescents did not have a visible way to alert others of their symptoms, they found it hard to communicate at times with others in a way that would facilitate understanding and empathy for their nausea.

*“It’s a little frustrating when your family doesn’t understand how you feel and so in that non-understanding, they tend to not feel as sympathetic to it.” (Emily)*

Mothers expressed significant frustration regarding finding a physician who could understand their daughters’ symptoms and suggest appropriate treatment. Mothers felt like physicians at times dismissed their daughters’ symptoms as a psychological problem without further exploring the etiology of the nausea through medical evaluations or tests. In the absence of a biomedical explanation, the suggestion of stress as a primary contributor by pediatricians was perceived as invalidating and dismissive.

*“We were told so many times, it’s all in her head by certain pediatricians, and I’m like, look, this child, it’s not in her head, this is going on, like even with her out of school, she’s still sick, so it’s not that she’s just stressed for school – no…she’s on homebound now, she’s still sick, so there’s something going on that needs to be addressed, but a lot of doctors will just push ‘em out of the way, and say deal with it whatever” (Julia’s mother)*

Although adolescents did not make direct statements regarding prior experiences with physician dismissal, many relayed positive experiences with the specialized nausea clinic, specifically the validation they received for their symptoms and focus on the whole person, both mind and body.

*“It felt good to know that someone was taking it seriously and giving me different ways to help cope with it instead of simply letting it happen and waiting for it to pass.” (Sarah)*

Overall, adolescents and their mothers desired for others to understand the severity and impact of nausea on their lives and respond with empathy and action. Specifically, adolescents desired others to understand when they were not feeling well, refrain from asking many questions, and accept them regardless of their symptoms. Mothers desired others to recognize the sacrifices they were making to care for their daughter at home and advocate for the best medical care possible.

### 3.4. Maternal Helplessness and Guilt

Because of the persistence of symptoms and significant impact of nausea on all domains of life, mothers often reported feeling helpless as to how to help their daughters. Mothers tried anything they could think of to alleviate their daughter’s nausea and were often left feeling frustrated when they were unable to help. For mothers who had also experienced chronic nausea, this feeling of helplessness was amplified by empathy.

*“I try to give her suggestions, things she could try…because I have nausea as well. So I try to help give her examples of what I’ve tried to do and what might help me, and see if it helps her...I felt helpless to help my daughter and it’s just I don’t know how to describe the emotion, but knowing what I’m going through and then to hear her say what I’m feeling has caused me a little stress because I feel bad in that I don’t know how to help because I haven’t solved it for myself.” (Emily’s mother)*

Feelings of helplessness often resulted from both sympathy (feeling bad for their daughter’s situation) and empathy (experiencing the same emotions as their daughter). Mothers often took on the emotional burden their daughters were experiencing and similarly felt helpless when nothing they tried to alleviate the nausea helped. Because mothers viewed themselves as primary caregivers who should be able to provide relief and care in this situation, they struggled when they were unable to fulfill this role in response to their daughters’ nausea.

*“[I am] constantly feeling bad for her because I know she wants to eat and can’t and like I said, as the mom, you want to be able to fix whatever’s wrong with your kid, when something makes them unhappy, when they’re not feeling well, then it makes you feel bad when there’s nothing you can do for it.” (Sarah’s mother)*

As the nausea persisted without significant relief, some mothers experienced less patience for their daughters’ situation or were unable to help to the same degree due to competing roles such as working a full-time job. Mothers reported feeling guilty when they experienced frustration or were unable to modify their schedule or behavior to accommodate their daughter’s nausea.

*“When my child calls me needing me to come get her and I know that it’s something she could ride out at school, I get a little perturbed and it’s just because I’m anxiety ridden about it. Like I can’t leave work all the time. I wish I could pick her up any time she’s uncomfortable but I can’t. A full-time working parent cannot do that.” (Ann’s mother)*

Mothers strongly desired to do anything they could to help alleviate their daughters’ discomfort. When their attempts were unsuccessful, mothers felt helpless. When mothers were unable to respond in the way they wanted to, they reported feeling guilty. Overall, mothers carried an emotional burden due to their daughters’ nausea, which often prompted behaviors such as doing anything they could to make their daughters feel more comfortable. At times, mothers shifted from this place of helplessness to a place of empowerment, recognizing that engagement in daily activities despite the nausea could be helpful for their daughters.

*“I say you’ve got to power through it.” (Louise’s mother)*

## 4. Discussion

Qualitatively evaluating the experiences of five adolescent girls with functional nausea and their mothers revealed four themes that captured the broad and largely negative impact nausea has had on adolescents and their mothers across multiple life domains. These four themes were labeled nausea interference, body frustration, misunderstanding of symptoms, and maternal helplessness and guilt. Across all themes, adolescents and their mothers struggled to make sense of their nausea and current life in relation to their life before nausea.

Adolescents described how nausea limited their functioning across multiple domains in their life, including physical, emotional, and social functioning. These experiences provide rich descriptions of the overall impact functional nausea can have on quality of life. Prior quantitative studies of youth with nausea support this theme. For example, youth with functional abdominal pain and chronic nausea, compared to those without nausea, reported a lower quality of life (referring to adolescent’s health and wellbeing), increased fatigue and decreased school attendance [10]. These findings are consistent with adolescents’ reports of social disruptions due to reduced school attendance, declines in mental health, and increased symptoms of anxiety in the current study. 

Adolescents with nausea and their mothers also expressed significant distress around the impact of nausea on eating behaviors and subsequent weight loss. For adolescents, this contributed to overall feelings of frustration and psychological distress regarding their body. Adolescents felt like their body was betraying them and working against them, keeping them from enjoying the life they desired. One adolescent described this phenomenon as feeling trapped and wanting to escape from her own body. Thus, the persistent experience of nausea appeared to contribute to heightened psychological distress due to negative cognitions about body functionality and the dissonance between adolescents’ desired functioning and their day-to-day experience.

Although a few studies have described a heightened prevalence of eating disorders in relation to chronic pain [30,31], eating behavior is one functional domain that could be disproportionately impaired by nausea compared to other somatic symptoms, such as pain. The thoughts and emotions adolescents with functional nausea described around their body image were generally atypical for adolescents with anorexia nervosa in that they did not express a desire for thinness. In fact, adolescents with nausea tended to report the opposite, a strong desire to eat more in order to gain weight as well as significant distress about nausea-related weight loss. The criteria for avoidant/restrictive food intake disorder (ARFID), a Diagnostic and Statistical Manual of Mental Disorders (DSM-5) diagnosis, however, clearly states a lack of body image concerns [32]. Many of the consequences of chronic nausea, such as weight loss, fear of negative results caused by eating, restriction diets, avoidance of foods, and difficulties with psychosocial aspects of daily living could meet criteria for ARFID. As with many psychological disorders, ARFID must not be better explained by another disorder, which is where differential diagnosis becomes difficult. A liberal definition of ARFID may capture these individuals with nausea who have lost a significant amount of weight and have psychosocial difficulties, while a more conservative definition would say that these symptoms are best explained by functional nausea, and therefore do not meet the criteria for ARFID. 

An important consideration regarding diagnostic conceptualization and treatment planning is that adolescents and mothers reported feeling misunderstood and dismissed when physicians suggested an eating disorder or psychological origin of their nausea. Thus, conceptualizing the impact of functional nausea as an eating disorder, such as ARFID, may hinder instead of enhance treatment engagement. “Diagnostic uncertainty” and “pain dismissal” are two concepts described in the pediatric chronic pain literature relevant to the experiences described by adolescents with functional nausea and their mothers [26,33]. Diagnostic uncertainty refers to the perceived lack of a diagnostic label or incorrect label for symptoms, whereas pain dismissal refers to the minimization and underestimation of the severity of symptoms. Both constructs have been associated with feelings of stigmatization and emotional distress in youth with chronic pain [26,33]. Adolescents with nausea in this study similarly described feeling either misunderstood or dismissed by friends and family due to the lack of visible indicators for their nausea. Additionally, one mother detailed her experience of being told that her daughter’s symptoms were “all in her head” and felt like her child’s nausea was not taken seriously by their child’s pediatrician. Pain and nausea share many characteristics including that they are both subjective in nature. Thus, these concepts of diagnostic uncertainty and dismissal likely apply across a range of chronic somatic symptoms and are not limited to chronic pain. 

In striving to reduce diagnostic uncertainty and pain dismissal, an integrated biopsychosocial approach to conceptualizing and treating functional nausea could prove useful. Mothers expressed a desire for clear medical diagnosis and treatment, and at the same time recognized the significant impact of nausea on adolescents’ mental health and social functioning. Similar to the fear-avoidance model of chronic pain [34], adolescents with functional nausea reported avoiding activities or foods that they feared would increase their nausea, which in turn led to increased anxiety, feelings of sadness, and lower levels of social engagement. Mothers appeared to reinforce this cycle by encouraging adolescents to avoid nausea triggers, planning meals based on adolescents’ preferred foods, and attending to adolescent symptoms due to their anxiety around their child’s suffering. In addition to evaluating and treating biological factors contributing to nausea, evaluating and treating these psychosocial factors could prove equally important for improving adolescents’ functioning and quality of life. 

Addressing the psychosocial components of functional nausea in pediatric patients likely requires a family-based approach. Mothers’ high levels of helplessness and guilt surrounding their child’s nausea and their own responses likely perpetuate a process called “miscarried helping.” Miscarried helping refers to the process where a caregiver inadvertently contributes to negative parent–child interactions surrounding health behaviors due to their strong desire to be helpful [35]. Mothers in this study expressed desires to keep their daughters from experiencing any discomfort, which is natural for any caregiver. Persistent somatic symptoms such as nausea, however, often require an approach that redirects parent attention away from symptoms and onto more adaptive coping behaviors their child displays that facilitate engagement in valued life activities [36]. Thus, attending to and accommodating symptoms of nausea due to increased caregiver distress may inadvertently increase adolescents’ functional impairment due to nausea. In the delivery of these interventions, it is likely important to emphasize to mothers what they can do to empower their child, instead of attending to symptoms, in order to reduce feelings of helplessness. Mothers expressed that they felt guilty when they ignored their child’s symptoms in the past; thus, psychoeducation and framing these interventions as a different approach to help their child regain function may reduce parental distress and increase parental adherence. Further, several mothers had also experienced chronic nausea themselves, which enhanced feelings of empathy and responsiveness to their daughter’s nausea. This is consistent with previous work more broadly related to the intergenerational transmission of risk for FGIDs [22] and somatic symptoms [37,38]. Evaluating intergenerational influences on the development and maintenance of pediatric functional nausea represents an area for future research.

The strengths of the current study included the qualitative focus on understanding the experience of adolescents with functional nausea and their mothers. Qualitative research allows for the development of a rich understanding of a construct without the limitations of traditional quantitative measures. In the present study, this led to further understanding themes specifically related to eating behaviors and cognitions surrounding the body that are not currently captured in quantitative studies of pediatric functional nausea. The study should also be evaluated in light of its limitations. Although the majority of our sample presented with functional nausea alone, two participants also had a history of irritable bowel syndrome which could have additionally influenced their quality of life. All adolescents, however, were referred to the specialty nausea clinic after an evaluation by a pediatric gastroenterologist because functional nausea was their primary symptom complaint. It is important to note that adolescents in this study were receiving specialized, integrated care for functional nausea which could have affected the lens through which they viewed their journey and symptoms. The experiences of this sample may not be generalizable beyond the timing and setting of this interview or to patients seen in primary care. Further, our sample is comprised of White female adolescents and their mothers. Thus, these findings cannot be extrapolated to male adolescents, fathers, or youth from other racial backgrounds. Other studies have had similar demographics in regards to racial makeup [10,14]. This could be due to a variety of factors such as who is more likely to initiate a medical complaint, the racial composition of the area from which the sample was taken, or other confounding factors; racial diversity was not used as part of the sampling approach in this study. 

## 5. Conclusions

The present study represents the first to our knowledge to qualitatively analyze the experiences of adolescent girls with functional nausea and their mothers. Mothers in this sample bore a great amount of emotional labor in caring for their daughters, advocating for the reality of their symptoms, and working through diagnostic uncertainty due to disbelief and misunderstanding from non-specialized physicians. Mothers shared feelings of guilt and frustration with their inability to help their daughters and eliminate their suffering due to nausea. Nausea impacted all aspects of the adolescent girls’ lives: interactions with family and friends around meal times, social interactions both inside and outside of school, mood symptoms, and eating patterns. Adolescent girls felt frustrated with their bodies’ inabilities and the discomfort that their bodies caused them. Many also experienced significant weight loss and displayed restrictive eating patterns as a result of the nausea. 

A common thread throughout the interviews, despite the narratives of discomfort and frustration, was optimism and an ability to hold hope for other families. In the midst of the difficulties that chronic nausea posed to these families, they maintained hope and encouraged other families to continue to fight to find a solution. Ultimately, this study illuminated the voices and experiences of adolescents with the recently formalized and under-investigated diagnosis of functional nausea, and their mothers. The themes and experiences highlighted will help inform clinical care and future research focused on improving the overall quality of life and medical experience of families coping with pediatric functional nausea.

## Figures and Tables

**Table 1:** Semi-Structured Qualitative Interview Schedule.

Interview Questions	
Adolescent	Mother
1. Tell me about your journey with nausea. a. Where did this all start? b. Where are you now in your journey? c. Where do you see this going?	1. Tell me about your journey with your teen’s nausea? a. Where did this all start? b. Where are you now in your journey? c. Where do you see this going?
2. How has nausea impacted your life?	2. What changes have you noticed in your teen since their nausea started?
3. How have friends and family responded to your nausea and the impact it has had on your life?	3. How do you tend to respond to your teen’s nausea?
	4. How has your teen’s nausea impacted your life?
